# Hydroxytyrosol and Olive-Derived Hydroxytyrosol-Rich Products for Skin Health and Prevention of Skin Ageing: Current Evidence and Future Perspectives

**DOI:** 10.3390/antiox15091095

**Published:** 2026-08-31

**Authors:** Adriana Albini, Danilo Morelli, Cecilia Bender, Paola Corradino

**Affiliations:** 1 Scientific Directorate, European Institute of Oncology (IEO), Istituto di Ricovero e Cura a Carattere Scientifico (IRCCS), 20141 Milan, Italy; 2 Fondazione IRCCS Istituto Nazionale dei Tumori di Milano, 20133 Milan, Italy; danilo.morelli@istitutotumori.mi.it; 3 Institut Kurz GmbH, Nattermannallee 1, 50829 Cologne, Germany; c.bender@kurz-italia.com; 4 European Institute of Oncology (IEO), Istituto di Ricovero e Cura a Carattere Scientifico (IRCCS), 20141 Milan, Italy; paola.corradino@ieo.it

**Keywords:** hydroxytyrosol, olive polyphenols, skin health, skin ageing, cancer, nutraceuticals, oxidative stress

## Abstract

Skin health is increasingly recognised as a key determinant of overall well-being, and its decline is driven by multiple interconnected biological processes. Hydroxytyrosol (HT), an olive polyphenol, exhibits pleiotropic biological activities and has a potential role in supporting skin homeostasis. This narrative review critically evaluates the evidence on oral HT and HT-rich olive-derived nutraceuticals for skin health and healthy ageing with a focus on oxidative damage, inflammation, photoprotection, extracellular matrix (ECM) preservation, wound healing, and skin pigmentation conditions. HT exhibits high oral bioavailability and modulates antioxidant defences, inflammatory signalling, autophagy, apoptosis, and metabolic homeostasis. Preclinical studies consistently show protective effects against oxidative stress, ECM degradation, cellular senescence, photoageing, impaired wound healing, and ultraviolet-induced skin damage. Experimental evidence also indicates antiproliferative and pro-apoptotic effects in melanoma models through modulation of oncogenic signalling pathways, suggesting a potential role in skin cancer prevention. In vivo, oral HT administration improves glycative skin ageing, psoriasis, intestinal barrier integrity, and systemic inflammation. Clinical evidence supports systemic antioxidant and cardiometabolic benefits of purified HT and HT-rich formulations, while dermatological evidence remains preliminary for psoriasis, melasma, and wound healing. Overall, HT shows promise, but randomised controlled trials with validated dermatological endpoints are needed to confirm its clinical efficacy.

## 1. Introduction

The skin is a dynamic, multifunctional organ essential for maintaining homeostasis through its roles in barrier function, thermoregulation, and immune surveillance, which contribute to overall health [1]. Skin health is closely associated with the integrity of the skin barrier, which provides protection against ultraviolet (UV) radiation, pollutants, and pathogens while preserving hydration, extracellular matrix (ECM) homeostasis, efficient wound healing, and protection from oxidative stress and inflammation [2,3,4]. These functions contribute to skin elasticity, resilience, and overall appearance [5] but are progressively compromised during ageing, leading to reduced skin hydration, impaired repair capacity, increased skin pH, and the development of both cutaneous and systemic inflammation [6,7]. Skin ageing results from a complex interplay of intrinsic and extrinsic factors, with oxidative stress and chronic low-grade inflammation representing the primary drivers of progressive structural and functional skin decline [8,9]. Additional contributors include poor nutrition, dehydration, sleep deprivation, hormonal changes, particularly the age-related decline in oestrogen, and alterations in the skin microbiome and gut–skin axis, which have emerged as important regulators of skin homeostasis, immune function, and age-related skin changes [10,11,12,13,14]. Because excessive reactive oxygen species (ROS) production can promote lipid peroxidation, protein oxidation, DNA damage, inflammatory signalling, and ECM remodelling, antioxidant and redox-modulating strategies are increasingly investigated as complementary approaches to support skin function during ageing [8,9].

These mechanisms have stimulated growing interest in systemic and dietary strategies aimed at supporting skin health and healthy ageing [5]. Among naturally occurring bioactive compounds, hydroxytyrosol (HT) is one of the major phenolic compounds naturally present in olives, extra virgin olive oil, and olive-derived by-products and has attracted considerable attention because of its potent antioxidant and anti-inflammatory properties [15,16]. HT (3,4-dihydroxyphenylethanol) is a low-molecular-weight ortho-diphenolic compound of the phenylethanoid class, occurring both in free form and as a hydrolysis product of oleuropein (OLE), the major secoiridoid glucoside of the olive tree [17]. It is present in olive fruits, virgin olive oil, and olive leaves, and it accumulates at particularly high concentrations in olive mill wastewater (OMWW), the aqueous by-product generated during olive oil extraction, which is particularly important in relation to sustainable, circular-economy-based formulation strategies [18]. The catechol structure of HT enables efficient scavenging of ROS while also modulating endogenous antioxidant and anti-inflammatory pathways [19,20,21,22,23,24]. In addition, HT has attracted increasing interest for its pleiotropic biological effects, including regulation of autophagy, mitochondrial function, ECM homeostasis, and immune responses [25,26]. These properties are relevant to antioxidant research because they connect a redox-active natural product with mechanisms of action, pharmacokinetic considerations, nutraceutical applications, and potential benefits for human health. Moreover, these mechanisms are closely involved in the pathogenesis of skin ageing and inflammatory skin disorders [27], making HT an attractive candidate for promoting skin health through nutritional approaches. Furthermore, the high oral bioavailability and favourable safety profile of HT have stimulated growing interest in its use as a nutraceutical for healthy ageing and disease prevention [28,29]. Indeed, oral HT supplementation has been associated with improvements in oxidative balance, inflammatory status, and metabolic health, all of which are relevant to skin homeostasis and ageing processes [30,31,32]. However, the dermatological relevance of oral HT remains incompletely defined. Available studies differ in experimental models, including cell-based systems, reconstructed skin models, ex vivo tissues, animal models, and human studies, as well as in HT source, formulation, dose, duration of exposure or supplementation, and outcome measures. While cellular and preclinical models are essential for elucidating mechanisms related to oxidative stress, inflammation, ECM remodelling, pigmentation, wound repair, and photoprotection, their translational value depends on confirmation in appropriately designed clinical studies, when feasible, using validated dermatological endpoints. Although several narrative reviews have summarized the biological activities of HT in the context of healthy ageing and chronic diseases, no review has specifically focused on the role of oral HT and HT-rich formulations in skin homeostasis and healthy ageing. This represents an important gap because the evidence linking HT to skin health is distributed across several areas, including redox biology, photoprotection, inflammation, ECM remodelling, wound repair, pigmentation disorders, melanoma biology, metabolic health, and gut–skin axis regulation. The present review integrates current knowledge on HT bioavailability, skin-specific molecular mechanisms, preclinical and clinical dermatological evidence, melanoma prevention, wound healing, and sustainable olive-derived formulations, providing a comprehensive overview of the emerging role of HT as a nutraceutical for skin health. It also highlights emerging areas of research, including the potential role of HT in the gut–skin axis and in mitigating menopause-associated skin changes. By integrating evidence on HT bioavailability, redox-modulating mechanisms, formulation-dependent effects, and dermatological outcomes, this review provides a focused and critical synthesis of the biological plausibility, current strength of evidence, limitations, translational challenges, knowledge gaps and research priorities for oral HT and HT-rich olive-derived products in skin health and healthy skin ageing.

## 2. Methods

The literature was identified through searches of PubMed/MEDLINE, Scopus, and Google Scholar, complemented by manual screening of the reference lists of relevant articles to identify additional publications.

The search focused on studies investigating HT in relation to skin biology, skin ageing, photoprotection, wound healing, oxidative stress, inflammation, ECM remodelling, cellular senescence, the gut–skin axis, and olive-derived nutraceutical formulations. Both experimental and clinical studies, together with relevant review articles, were included to provide a comprehensive overview of the available evidence. Publications written in English and available up to June 2026 were included.

The selection of studies was based on their scientific relevance to the objectives of the review, with particular emphasis on investigations addressing the biological mechanisms, bioavailability, pharmacokinetics, preclinical efficacy, clinical evidence, and translational potential of HT for skin health. As this is a narrative review, the literature was synthesized qualitatively, and no formal systematic review methodology or meta-analysis was performed.

## 3. Skin Ageing and Alterations in Skin Homeostasis

Clinically, skin ageing is characterized by wrinkles, pigmentation changes, xerosis, epidermal thinning, actinic keratoses, and reduced dermal elasticity. Importantly, the deterioration of skin structure and function extends beyond cosmetic concerns, affecting barrier integrity, immune competence, and susceptibility to disease [33]. Skin ageing is a multifactorial process driven by both intrinsic (chronological) and extrinsic factors, including environmental and lifestyle influences, collectively referred to as the exposome. The exposome plays a major role in accelerating skin ageing, highlighting the need for preventive and targeted interventions [34]. The skin is highly vulnerable to environmental stressors such as ionising radiation, pollution, and UV exposure, as well as to lifestyle factors including alcohol consumption and poor diet [35]. In particular, UV radiation damages the dermal ECM and keratinocyte DNA, leading to structural and cellular alterations that, besides resulting in reduced skin elasticity and mechanical strength, impaired wound healing, and disrupted skin barrier function, also contribute to immunosuppression [35]. Intrinsic ageing, by contrast, is an inevitable process driven by genetic and metabolic factors [36].

Ageing is also associated with alterations in the skin microbiome and immune function, which may contribute to barrier dysfunction, chronic inflammation, and impaired tissue repair [37]. In women, menopause further accelerates skin ageing through oestrogen decline, resulting in reduced collagen synthesis, impaired elasticity, decreased hydration, thinning of the epidermis and dermis, and delayed wound healing [38]. Both extrinsic and intrinsic factors contribute to ageing by increasing ROS production and activating inflammatory pathways [39]. ROS accumulation promotes ECM degradation, collagen breakdown, cellular senescence, and progressive loss of skin function, contributing to the visible signs of ageing. Moreover, skin barrier dysfunction is increasingly recognised as a potential driver of systemic inflammageing, a chronic low-grade inflammatory state associated with systemic consequences [40,41]. This process involves glycation, reduced autophagy, mitochondrial dysfunction, telomere shortening, immune dysregulation, and diminished regenerative capacity [40,42,43,44].

Beyond its role in physiological ageing, redox imbalance also contributes to the pathogenesis of inflammatory skin disorders. In psoriasis, for example, it promotes keratinocyte hyperproliferation and immune activation, establishing a self-perpetuating cycle between oxidative stress and inflammation [45]. UV radiation and other environmental stressors further amplify oxidative damage through lipid peroxidation, protein oxidation, and DNA injury in keratinocytes and fibroblasts [8,9]. These alterations impair cellular function, accelerate cellular senescence, and increase matrix metalloproteinase (MMP) expression, ultimately promoting ECM degradation [46,47]. Sustained activation of inflammatory pathways also results in overproduction of pro-inflammatory cytokines, including tumour necrosis factor-α (TNF-α), interleukin (IL)-1β, and IL-6, further exacerbating tissue damage [48,49]. In parallel, ageing of the skin immune system reduces pathogen defence, impairs clearance of senescent cells, and promotes dysregulated inflammatory responses, thereby increasing susceptibility to infections, inflammatory disorders, and delayed wound healing [43].

Among the intrinsic drivers of ageing, advanced glycation end products (AGEs) have attracted considerable attention. AGEs are formed through non-enzymatic reactions between reducing sugars and macromolecules and originate from both endogenous metabolism and dietary sources [50,51]. In the skin, AGEs accumulate in collagen and elastin fibers, increasing stiffness, reducing elasticity, and disrupting ECM turnover [52,53,54]. They also promote fibroblast apoptosis, reduce collagen content, and enhance dermal fragmentation [55]. Furthermore, AGEs contribute to oxidative stress and inflammation by increasing ROS production and inducing mitochondrial dysfunction [56]. Their biological effects are mediated through protein crosslinking, ROS generation, and activation of receptor-mediated pathways, particularly the receptor for advanced glycation end products (RAGE), which triggers nuclear factor kappa-light-chain-enhancer of activated B (NF-κB)-dependent inflammatory signalling and cytokine production [56,57]. Although autophagy normally contributes to AGE clearance, excessive glycation impairs autophagic function, creating a vicious cycle that promotes further AGE accumulation and cellular dysfunction [58,59]. Conversely, activation of autophagy has been shown to facilitate AGE removal and improve cellular homeostasis [60].

The gut–skin axis is increasingly recognised as a key contributor to skin ageing and inflammatory skin disorders through the interplay between the gut microbiota, immune responses, and circulating metabolites [14]. Among the mechanisms underlying this connection, oxidative stress appears to play a central role. Increased ROS levels can impair intestinal barrier integrity, promoting microbial translocation and systemic inflammatory responses. In turn, gut dysbiosis may further enhance redox imbalance through immune dysregulation and the production of pro-inflammatory metabolites, creating a self-perpetuating cycle that contributes to chronic inflammation, disruption of tissue homeostasis, and progression of inflammatory skin conditions [61,62].

Increasing evidence highlights the importance of preserving skin barrier integrity, reducing oxidative stress, and modulating inflammation not only to maintain skin health but also to mitigate systemic ageing processes [63]. Consequently, plant-derived phytochemicals are increasingly being explored as therapeutic and preventive strategies for inflammatory skin conditions, both as topical agents and nutritional supplements. These compounds may modulate inflammatory pathways, provide photoprotective effects, and support skin barrier function with fewer adverse effects than conventional pharmacological approaches [64,65,66,67]. Among these bioactive compounds, HT has emerged as a particularly promising candidate due to its combined antioxidant, anti-inflammatory, and metabolic effects, which target several of the biological pathways involved in skin ageing and skin homeostasis.

## 4. Hydroxytyrosol: Bioavailability and Mechanistic Pathways

### 4.1. HT Bioavailability

HT displays excellent oral bioavailability and, following ingestion, is efficiently absorbed in the small intestine and rapidly metabolized into glucuronide and sulphate conjugates that retain significant antioxidant and anti-inflammatory activity [28,29]. Peak plasma concentrations are generally reached within 30–60 min after administration, supporting the rapid onset of systemic biological effects observed in acute intervention studies [68]. HT bioavailability is influenced by several factors, including formulation, lipid matrix, dose, timing of administration, and the presence of other polyphenols [69,70]. These characteristics make HT particularly suitable for oral supplementation strategies aimed at targeting systemic pathways involved in oxidative stress, inflammation, and tissue homeostasis.

### 4.2. HT Mechanistic Pathways

Mechanistically, HT modulates multiple interconnected pathways involved in cellular homeostasis, extending beyond antioxidant and anti-inflammatory activities to include metabolic regulation and microbiota-modulating effects [71,72]. Rather than acting solely as a direct scavenger of ROS, HT primarily functions as a regulator of endogenous cellular defence systems. A central mechanism involves activation of the nuclear factor erythroid 2-related factor 2 (Nrf2) signalling pathway. By upregulating the expression and activity of antioxidant and detoxifying enzymes, including glutathione peroxidase, glutathione reductase, and glutathione S-transferase, Nrf2 promotes the protection of cells against oxidative stress-induced damage [73].

The anti-inflammatory activity of HT is mediated through the inhibition of multiple endogenous inflammatory mediators and signalling pathways, including NF-κB and cyclooxygenase-2 (COX-2). HT also prevents the degradation of inhibitor of nuclear factor kappa B (IκB), suppressing NF-κB nuclear translocation and mitogen-activated protein kinase (MAPK)/p38 MAPK activation [74]. These effects result in reduced production of pro-inflammatory mediators such as C-reactive protein (CRP), TNF-α, IL-1β, and IL-6, together with increased expression of anti-inflammatory mediators including IL-10 and IL-4 [21,22,23,75]. Moreover, HT promotes macrophage polarisation from the pro-inflammatory M1 phenotype toward the anti-inflammatory M2 phenotype, thereby contributing to the resolution of inflammation and tissue protection [75]. Clinical studies further support the broad anti-inflammatory activity of HT [76].

Growing evidence also indicates that HT influences cellular quality-control mechanisms involved in ageing and tissue homeostasis. In particular, HT has been shown to regulate apoptosis, autophagy, and immune responses while interacting with lipid metabolism and epigenetic signalling pathways [71]. In primary rat chondrocytes exposed to oxidative and inflammatory stress, HT enhanced autophagic activity through a Sirtuin 1 (SIRT1)-dependent mechanism, increasing the expression of key autophagy-related proteins, including microtubule-associated protein 1 light chain 3-II (LC3-II) and autophagy-related protein (ATG) 5/7, while reducing p62 accumulation. These effects were accompanied by reductions in ROS production, NADPH oxidase (NOX)2 and NOX4 expression, and inflammatory mediators including IL-6, TNF-α, and MMP-13, ultimately improving cellular viability under stress conditions [77]. Importantly, inhibition of either autophagy or SIRT1 abolished these protective effects, confirming the central role of this pathway.

Beyond intracellular signalling, HT exerts beneficial systemic effects that are highly relevant to ageing-related processes. Cardiovascular protection represents one of the most extensively documented areas of research. HT improves endothelial function, enhances nitric oxide bioavailability, reduces low-density lipoprotein (LDL) oxidation, and attenuates inflammatory signalling, thereby contributing to improved vascular homeostasis and reduced cardiovascular risk [78].

Emerging evidence also suggests that HT may influence microbiota composition and gut barrier function, contributing to modulation of the gut–skin axis and systemic inflammatory responses [72]. These activities are particularly relevant in the context of skin ageing, where redox imbalance, chronic inflammation, metabolic dysfunction, and microbiome alterations interact to drive progressive deterioration of skin structure and function.

Overall, HT displays a favourable safety profile and appears particularly effective under conditions characterized by increased oxidative stress and chronic inflammation [79]. Through the coordinated modulation of antioxidant defences, inflammatory pathways, cellular quality-control mechanisms, and systemic metabolic processes, HT targets several of the biological mechanisms implicated in skin ageing and impaired skin homeostasis.

### 4.3. Systemic Effects of Hydroxytyrosol-Rich Formulations and Purified Hydroxytyrosol

**Figure 1:**
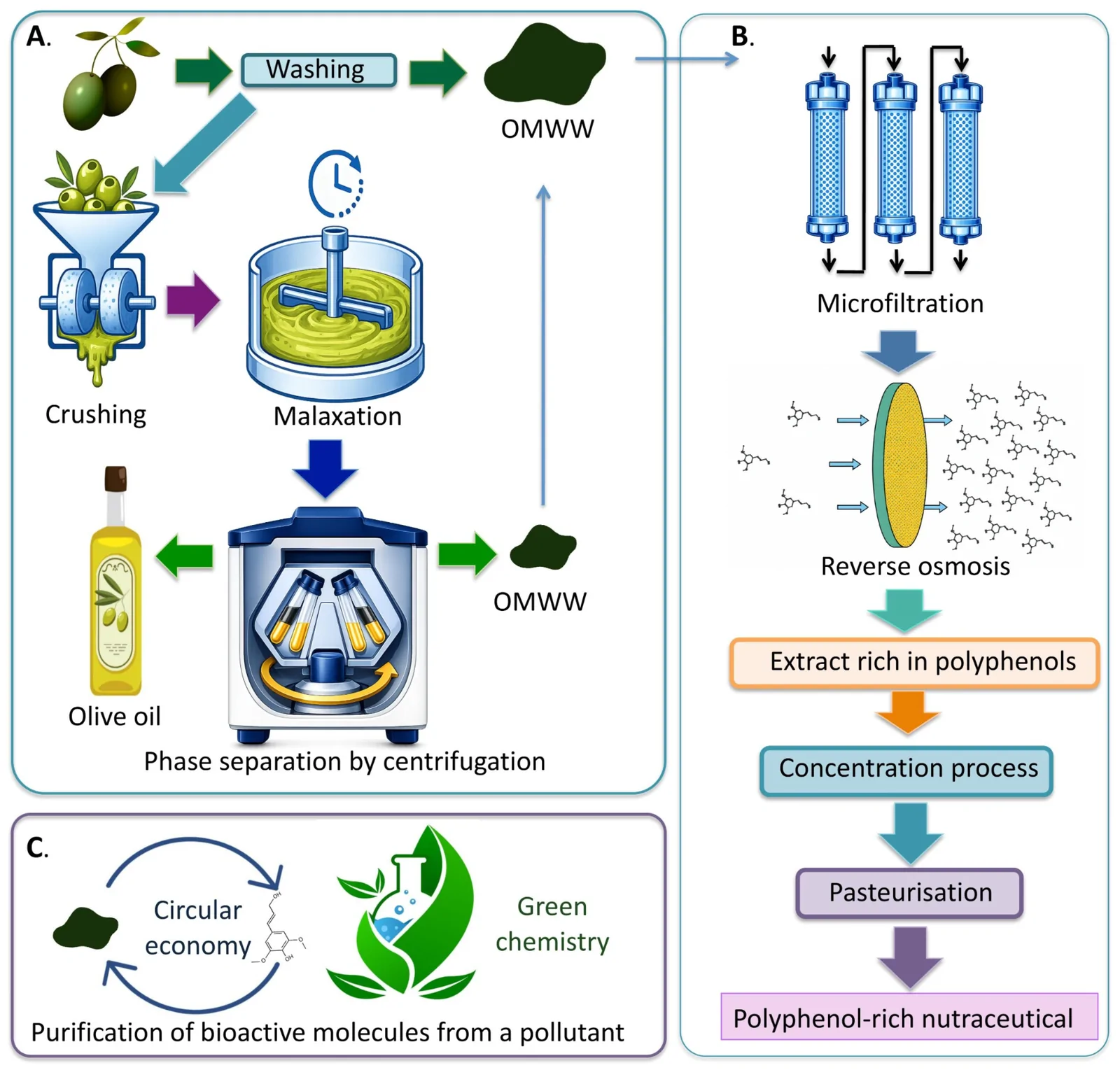
Recovery of HT-rich fractions from OMWW through a circular economy approach. (**A**) During extra virgin olive oil production, olives undergo washing, crushing, malaxation, and centrifugation, generating both olive oil and OMWW as a by-product. (**B**) OMWW is subsequently processed through a series of steps, including microfiltration, reverse osmosis, concentration, and pasteurization, resulting in the recovery of a polyphenol-rich extract. (**C**) This process exemplifies a circular economy and green chemistry strategy, whereby a potentially polluting agricultural waste stream is transformed into a valuable source of bioactive compounds with nutraceutical applications.

Olive oil contains a variety of bioactive constituents that can be broadly classified into two fractions: the unsaponifiable (non-polar) fraction, which includes squalene, triterpenes, sterols, tocopherols, and pigments, and the polar fraction, which comprises phenolic compounds, including HT [80]. In addition to olive oil, HT and other phenolic compounds are highly concentrated in OMWW, the aqueous by-product generated during extra virgin olive oil production. Despite being biodegradable, OMWW poses significant environmental challenges. Its high phenolic content and acidic pH contribute to phytotoxic effects, while its lipid-rich composition can reduce dissolved oxygen levels, adversely affecting aquatic ecosystems [81]. Advances in extraction and purification technologies have enabled the recovery of polyphenol-rich fractions from OMWW, transforming an agricultural waste stream into a valuable and sustainable source of nutraceutical compounds within a circular economy framework [82]. One example is OliPhenolia^®^, a phytocomplex obtained from OMWW through reverse osmosis, concentration, and mechanical filtration processes. HT is its most abundant phenol, accounting for approximately 18.3% of the total phenolic content (82.50 mg/50 mL), followed by verbascoside (Verb; 7.2%; 32.50 mg/50 mL), tyrosol (2.3%; 10.50 mg/50 mL), p-coumaroyl secoiridoids (1.4%; 6.50 mg/50 mL), and other minor phenolic compounds. Consequently, the biological activity of OliPhenolia^®^ is likely to result from the combined action of multiple bioactive constituents rather than from HT alone [83,84]. The preparation process of OMWW-derived extracts is depicted in Figure 1.

#### 4.3.1. Evidence from HT-Rich Formulations

The first human studies investigating OMWW-derived formulations primarily focused on bioavailability and antioxidant activity. In a pilot crossover study involving healthy volunteers, Bender et al. demonstrated that HT-rich formulations derived from OMWW were rapidly absorbed after oral administration, as evidenced by the urinary excretion of HT. In parallel, in vitro experiments showed significant antioxidant activity, including reductions in ROS, lipid peroxidation, and AGE formation, together with stimulation of endogenous antioxidant defences [85].

The bioavailability of the formulation was confirmed in a randomised, double-blind, placebo-controlled study conducted in healthy recreationally active adults. Following ingestion of a single dose, plasma HT concentrations increased rapidly and remained elevated for several hours. During a subsequent exercise challenge designed to induce oxidative stress, supplementation resulted in modest but significant antioxidant effects, including increased glutathione levels and reduced superoxide dismutase activity following exercise, suggesting improved redox balance and recovery capacity. However, no significant effects were observed for several other oxidative stress biomarkers, indicating that the physiological impact was relatively modest [86].

A more detailed pharmacokinetic characterization of olive-derived HT formulations was subsequently provided by Bender et al. in a randomised, controlled, blinded crossover study in 12 healthy volunteers. Using serial blood and urine sampling over 12 h after a single dose, the authors tracked both free HT and its major metabolites in plasma and urine, showing rapid absorption, a peak plasma concentration at approximately 30 min, dose-dependent bioavailability, and extensive phase II/metabolic conversion. By integrating plasma kinetics with urinary excretion data, this study provided a mechanistic basis for subsequent efficacy analyses in the same experimental setting [87]. Additional evidence for short-term antioxidant activity was provided by the analysis of biomarkers of lipid oxidation, including plasma oxidized LDL (oxLDL) and urinary F2-isoprostanes, which showed a reduction following supplementation. Interestingly, these effects appeared more pronounced in participants with higher baseline oxidative stress, suggesting that the benefits of supplementation may be greater under conditions of increased oxidative burden [88]. Although the study was designed around cardiovascular markers rather than dermatological endpoints, these findings remain of interest in a beauty-from-within context because F2-isoprostanes are robust markers of in vivo lipid peroxidation [89,90], while oxidative stress-related lipid damage has also been documented in skin under oxidative challenge and is implicated in skin ageing and age-related tissue damage [8].

Beyond antioxidant activity, HT-rich formulations have shown promising effects on metabolic health. In a pilot longitudinal intervention study involving 29 adults presenting characteristics associated with metabolic syndrome, supplementation with the OMWW preparation OliPhenolia^®^ (25 mL twice daily for 30 days) was associated with favourable changes in several cardiometabolic parameters, including reductions in blood pressure, fasting glucose, insulin levels, and LDL cholesterol. Improvements were also observed in hydration status, while metabolomic analyses suggested modulation of pathways related to vitamin D metabolism, homocysteine metabolism, and inflammation. Importantly, no clinically relevant adverse effects were reported [91]. A secondary analysis of the same cohort further explored body composition and muscle-related outcomes. Supplementation was associated with modest increases in skeletal muscle mass and muscle mass percentage, reductions in fat mass, and improvements in hydration status. These changes were accompanied by increased antioxidant capacity, reflected by higher protein thiol concentrations and Trolox equivalent antioxidant capacity, suggesting a potential role for OMWW-derived polyphenols in supporting muscle health and counteracting early sarcopenic changes in individuals at metabolic risk [84].

Evidence supporting broader cardiometabolic benefits also comes from studies using HT-enriched foods. In a 12-week randomised dietary intervention including 60 adults with overweight/obesity and type 2 diabetes, daily consumption of HT-enriched whole wheat bread within an energy-restricted Mediterranean diet produced greater reductions in fasting glucose, glycated haemoglobin (HbA1c), insulin levels, LDL cholesterol, body fat, and inflammatory markers, particularly TNF-α, compared with conventional whole wheat bread [92].

Similarly, a 6-month pilot intervention study conducted in 11 healthy older adults evaluated the effects of olive oils differing in polyphenol content. Participants consuming high-polyphenol extra virgin olive oil, characterized by higher concentrations of HT and tyrosol, showed reductions in arterial inflammation and atherosclerotic microcalcification assessed by positron emission tomography/computed tomography (PET/CT) imaging. In contrast, standard extra virgin olive oil produced no significant changes, while refined olive oil was associated with worsening microcalcification markers. These findings suggest a potential vascular protective effect of olive phenols in ageing populations [93].

#### 4.3.2. Evidence from Purified Hydroxytyrosol

Compared with HT-rich formulations, clinical evidence specifically evaluating purified HT remains more limited. Nevertheless, available studies generally support the biological effects observed with complex formulations. The strongest evidence currently derives from a randomised, double-blind, placebo-controlled trial involving 49 adults aged 40–70 years with overweight and prediabetes. Participants received either 15 mg/day of purified HT or placebo for 16 weeks. HT supplementation significantly reduced several biomarkers of oxidative stress and inflammation, including oxLDL, protein carbonyls, 8-hydroxy-2′-deoxyguanosine (8-OHdG), and IL-6, while preserving antioxidant capacity and glutathione peroxidase activity. These findings indicate that HT may be particularly effective under conditions characterized by increased oxidative stress and low-grade inflammation [31].

Overall, current human evidence consistently supports antioxidant, anti-inflammatory, and cardiometabolic effects of both HT-rich formulations and purified HT. However, most studies have evaluated systemic biomarkers and metabolic outcomes rather than dermatological endpoints. Therefore, while these findings provide a strong biological rationale for a potential role of oral HT supplementation in skin health, direct clinical evidence linking HT intake to improvements in skin ageing, skin function, or other dermatological outcomes remains limited.

## 5. Hydroxytyrosol and Skin Biology: Mechanistic Rationale and Implications for Topical and Oral Supplementation

The systemic biological activities of HT provide a strong mechanistic basis for its potential role in skin health. Because the molecular pathways involved in skin ageing largely overlap with those modulated by HT, including oxidative stress, inflammatory signalling, ECM remodelling, and impaired tissue repair, growing experimental evidence has investigated its direct effects on skin biology [52,94].

The integrity of the ECM is a major determinant of skin structure, elasticity, and mechanical resilience. HT contributes to ECM homeostasis by limiting collagen degradation, reducing MMP activity, and supporting collagen preservation. Through these effects, HT may help maintain skin firmness and counteract structural alterations associated with chronological ageing and photoageing [95,96].

UV exposure accelerates ECM degradation and skin ageing by amplifying oxidative stress and inflammatory signalling. By modulating these pathways, HT may help preserve skin architecture and function while limiting photoageing-associated damage [97].

Beyond its anti-ageing properties, HT appears to support tissue repair and regenerative processes. Experimental studies indicate that HT promotes fibroblast proliferation and migration, enhances re-epithelialisation, stimulates ECM remodelling, and supports wound closure. In addition, HT modulates epithelial–mesenchymal transition (EMT) and inflammatory responses involved in tissue repair, potentially facilitating effective healing while limiting excessive fibrosis and pathological remodelling [98,99]. Evidence also suggests that HT activates cytoprotective pathways enhancing cellular resilience and repair capacity under conditions of oxidative stress through heme oxygenase-1 (HO-1)/Nrf2 signalling [100].

Collectively, these findings provide the biological rationale for both topical and oral applications of HT. Topical formulations may exert direct local effects on epidermal barrier function, inflammation, and tissue repair, whereas oral supplementation may indirectly support skin health by modulating systemic oxidative stress, inflammation, and metabolic homeostasis [52,101]. Although experimental evidence consistently supports these mechanisms, clinical studies evaluating dermatological outcomes remain limited and heterogeneous.

### 5.1. Studies Relevant to Skin Biology

The proposed mechanisms are supported by evidence from in vitro studies, animal models, and a limited number of clinical investigations. Because the biological effects of purified HT may differ from those of HT-rich olive-derived formulations containing additional bioactive polyphenols, the available evidence is presented separately according to study design and intervention. This distinction is important because complex olive-derived preparations contain several bioactive polyphenols that may act synergistically, potentially enhancing antioxidant, anti-inflammatory, and regenerative responses. To facilitate interpretation, the evidence is presented according to the level of investigation, moving from in vitro studies to animal models and, finally, clinical studies evaluating skin-related outcomes.

#### 5.1.1. In Vitro Evidence

A substantial body of in vitro evidence supports the protective effects of HT against oxidative stress, inflammation, ECM degradation, and cellular senescence (Table 1).

Studies using fibroblast and keratinocyte models consistently indicate that HT may counteract processes associated with skin ageing and environmental damage. In photoageing models, HT reduced UVA-induced cellular senescence and inflammatory responses, while also limiting ECM-degrading activity [95,97]. Other studies demonstrated antioxidant and anti-enzymatic effects, including inhibition of collagenase and elastase, with some evidence that combinations of HT with other olive phenols may enhance cytoprotective activity [102]. Chronic low-dose exposure further suggested a role for HT in delaying replicative senescence and attenuating the senescence-associated secretory phenotype (SASP) [96].

Anti-inflammatory effects have also been demonstrated in keratinocyte models. HT and related derivatives attenuated cytokine-driven inflammatory responses, in part through modulation of NF-κB signalling, and reduced keratinocyte hyperproliferation under psoriasis-like conditions [94,103]. These findings suggest that HT may influence both inflammatory signalling and epidermal homeostasis.

In addition to these protective effects, several studies support a regenerative role for HT. Experimental wound-healing models showed enhanced fibroblast proliferation and migration, improved wound closure, and modulation of pathways involved in tissue remodelling and repair [98,99,100]. In particular, activation of the Nrf2/HO-1 axis appears to contribute to cytoprotection and reparative responses, while modulation of EMT-related processes may help balance effective repair with the prevention of excessive fibrotic remodelling [98,100].

Overall, the in vitro evidence suggests that HT acts through multiple, interconnected mechanisms rather than a single molecular target. Its effects encompass redox regulation, suppression of inflammatory signalling, preservation of ECM integrity, delayed cellular senescence, and promotion of tissue repair. However, interpretation remains limited by the marked heterogeneity in cell models, concentrations, exposure times, and experimental stimuli, as well as by the frequent use of immortalized or non-cutaneous cells. These methodological differences should be considered when extrapolating the findings to human skin physiology (Table 1).

#### 5.1.2. In Vivo Evidence

Although more limited than the in vitro literature, available animal studies provide complementary evidence that HT may exert beneficial effects on skin structure, inflammation, and tissue repair under more complex biological conditions (Table 1).

In a model of AGE-induced skin ageing, oral HT improved several structural and functional parameters of the skin while also attenuating oxidative and inflammatory alterations [52]. Notably, the study also reported restoration of intestinal barrier markers, suggesting that systemic mechanisms, including modulation of the gut–skin axis, may contribute to the observed effects.

Protective activity has also been demonstrated in inflammatory skin disease. In an imiquimod-induced psoriasis-like model, HT reduced clinical and histological signs of disease and attenuated inflammatory signalling, with inhibition of ERK and NF-κB pathways emerging as relevant mechanisms [104]. These findings are consistent with the anti-inflammatory effects observed in keratinocyte models and support the potential relevance of HT in conditions characterized by epidermal hyperproliferation and immune activation.

The regenerative potential of HT is further supported by diabetic wound-healing experiments, in which treatment improved wound repair and promoted a more favourable inflammatory and reparative microenvironment [105]. Together, these data suggest that HT may influence multiple stages of tissue recovery, including inflammatory resolution, fibroblast activity, collagen deposition, and macrophage polarisation.

Taken together, the available in vivo findings reinforce the multifunctional profile observed in vitro and indicate that HT may act at both local and systemic levels. Nevertheless, the evidence is still based on a small number of animal studies using distinct disease models, routes of administration, and dosing regimens. Therefore, although these studies strengthen the biological plausibility of HT as a skin-protective agent, direct translation to humans remains premature.

#### 5.1.3. Preliminary Clinical Evidence

Clinical evidence remains substantially more limited than the preclinical literature and is currently restricted to a small number of pilot studies using HT-containing formulations (Table 1).

Preliminary findings are most encouraging in inflammatory skin disorders. In patients with mild-to-moderate psoriasis, an HT-enriched olive polyphenol nutraceutical was associated with improvements in clinical disease severity, suggesting a possible adjunctive role for olive-derived phenolic supplementation [106]. However, the formulation contained several bioactive components, preventing the observed effects from being attributed specifically to HT.

A second pilot study investigated oral and topical HT-containing preparations in women with melasma [107]. Although some improvements were observed, particularly with oral treatment, between-group differences were not statistically significant. These findings therefore suggest possible activity but do not provide definitive evidence of efficacy.

Overall, current clinical data should be considered preliminary. Small sample sizes, short follow-up, heterogeneous formulations, concomitant treatments, and the use of multi-component products all limit interpretation and make it difficult to determine the independent contribution of HT. Future trials should therefore employ standardised HT preparations, clearly defined doses and routes of administration, adequate sample sizes, and clinically relevant endpoints. Such studies will be essential to establish whether the antioxidant, anti-inflammatory, anti-ageing, and regenerative effects consistently observed in preclinical models translate into meaningful benefits in human skin.

**Figure 2:**
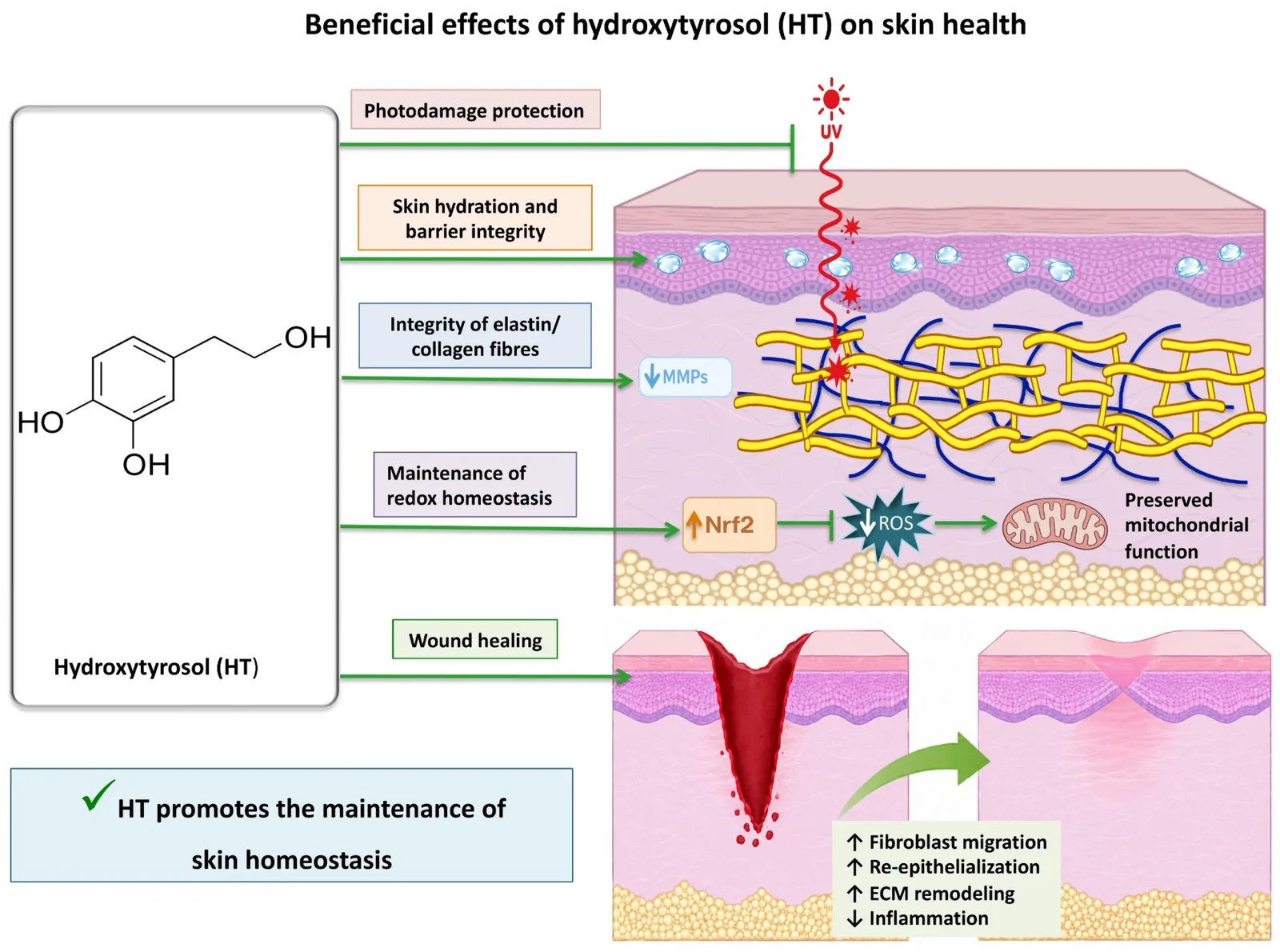
Schematic representation of the proposed effects, derived from mechanistic and preclinical evidence, of HT on skin health. HT contributes to the maintenance of skin homeostasis by supporting epidermal hydration, protecting against UV-induced damage, preserving the integrity of ECM components, including collagen and elastin fibers, and promoting tissue repair and wound healing. ECM, extracellular matrix; HT, hydroxytyrosol; MMPs, matrix metalloproteinases; Nrf2, nuclear factor erythroid 2-related factor 2; ROS, reactive oxygen species; UV, ultraviolet.

**Figure 3:**
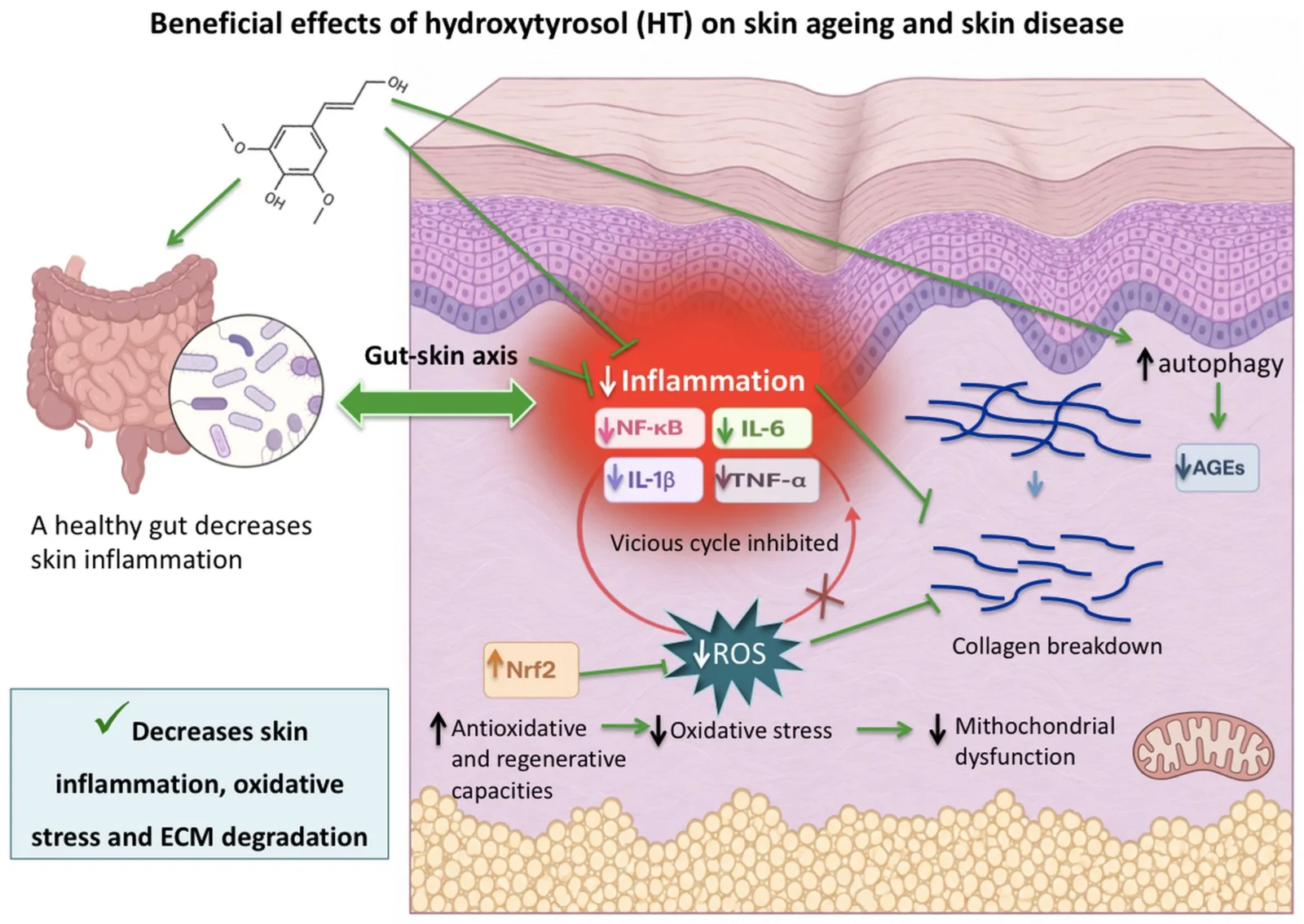
Proposed mechanisms, derived from mechanistic and preclinical evidence, by which HT may counteract skin ageing and inflammatory skin disorders. HT modulates multiple biological pathways involved in skin homeostasis, including the reduction in oxidative stress and ROS generation, attenuation of pro-inflammatory signalling pathways (e.g., NF-κB) and cytokine production (e.g., IL-1β, IL-6, and TNF-α), and enhancement of endogenous antioxidant defences through activation of Nrf2-dependent pathways. HT may also preserve extracellular matrix integrity by limiting collagen degradation, promote autophagy-mediated clearance of AGEs, and support mitochondrial function. In addition, emerging evidence suggests that HT may influence the gut–skin axis by improving intestinal barrier function and modulating microbiota-related inflammatory responses, thereby contributing to the maintenance of skin homeostasis and reducing age-related skin deterioration and inflammation. AGEs, advanced glycation end products; ECM, extracellular matrix; HT, hydroxytyrosol; IL, interleukin; NF-κB, nuclear factor kappa B; Nrf2, nuclear factor erythroid 2-related factor 2; ROS, reactive oxygen species; TNF-α, tumour necrosis factor alpha.

The proposed main mechanisms underlying the beneficial effects of HT and HT-enriched formulations on skin homeostasis and ageing are schematically illustrated in Figure 2 and Figure 3.

Collectively, available evidence indicates that HT exerts pleiotropic protective effects across multiple experimental models by targeting interconnected pathways involved in redox regulation, inflammation, extracellular matrix remodelling, photoprotection, and tissue repair. However, direct clinical evidence remains limited and is largely restricted to small pilot studies and specific dermatological conditions.

Beyond skin ageing and inflammatory skin disorders, growing evidence indicates that the antioxidant and anti-inflammatory mechanisms underlying the beneficial effects of HT may also contribute to protection against UV-induced photodamage and skin carcinogenesis. The following section summarizes the emerging preclinical evidence supporting the role of HT in photoprotection and skin cancer prevention.

## 6. Hydroxytyrosol: Emerging Evidence on Photoprotection and Skin Cancer Prevention

UV radiation is the principal environmental factor responsible for skin photoageing and a major contributor to skin carcinogenesis. Both UVA and UVB induce excessive production of ROS, leading to oxidative stress, DNA damage, chronic inflammation, activation of MAPK, NF-κB, and activator protein-1 (AP-1) signalling pathways, ECM degradation through increased MMP expression, and dysregulation of cell survival and apoptotic pathways. Together, these molecular events promote photodamage, accelerate skin ageing, and contribute to skin cancer development [108]. Consequently, compounds capable of limiting UV-induced cellular damage may also contribute to skin cancer prevention. HT has attracted considerable attention because, besides its antioxidant and anti-inflammatory properties, it exerts photoprotective activities and direct anticancer effects, providing the biological rationale for investigating HT as a multifunctional agent for both photoprotection and skin cancer prevention [109,110].

### 6.1. Photoprotective Effects

HT has emerged as a potential photoprotective agent, with preclinical evidence indicating that it can counteract several molecular processes involved in UV-induced skin damage and photoageing (Table 2).

In keratinocyte models, HT attenuated UVB-induced oxidative and genotoxic damage and modulated stress-related signalling, supporting a direct protective effect against UV-mediated cellular injury [109]. Similar findings were obtained following UVA exposure, although the magnitude of protection appears to depend on the chemical form of HT. In particular, a biocatalytically synthesized HT dimer showed greater antioxidant and anti-apoptotic activity than monomeric HT, suggesting that structural modification may enhance its photoprotective properties [97].

More recent evidence indicates that combining HT with other olive-derived phenolics may further enhance these effects. The association of HT with OLE and Verb provided broader protection against UVB-induced photoageing than the individual compounds, simultaneously targeting oxidative stress, inflammatory signalling, cellular senescence, apoptosis, and ECM degradation. Modulation of the MAPK/NF-κB and Nrf2 pathways appears to contribute to these effects, suggesting complementary actions of olive phenols on stress-response and antioxidant mechanisms [111].

Importantly, the photoprotective potential of HT has also been explored using a transdermal microneedle delivery system. This approach extended the findings from cellular models to experimental photoageing in vivo, supporting antioxidant, anti-senescent, and ECM-preserving effects [112]. Beyond providing further evidence of HT activity, this study highlights the importance of the delivery strategy, particularly considering the limitations associated with HT stability, bioavailability, and effective tissue exposure.

Overall, the available evidence suggests that HT may protect against UV-induced skin damage through interconnected antioxidant, anti-inflammatory, anti-apoptotic, and ECM-preserving mechanisms. However, most evidence remains preclinical, and considerable heterogeneity in experimental models and treatment conditions limits direct comparison between studies. Further research is therefore required to determine whether these effects can be reproduced at clinically relevant exposures and translated into effective photoprotective interventions.

**Table 2:** Preclinical evidence supporting the photoprotective and skin cancer preventive effects of hydroxytyrosol (HT) and HT-rich formulations.

Type of Study and Intervention	Concentration/Dose	HT/Intervention Treatment Time	Model	Stimulus/Exposure Conditions	Main Findings	Limitations	Reference
In vitro; pure HT	100–1000 μM	18 h pretreatment before UVA	M14 human melanoma cells	UVA emission spectrum peaked at 365 nm 7.5 J/cm^2^ for 4 h	↓ ROS (~40% at 400 μM) ↓ lipid peroxidation (TBARS) ↓ UVA-induced protein damage (↓ L-isoaspartyl residues) ↓ proliferation ↑ apoptosis and caspase-3 at ≥600–1000 μM	Melanoma cell model; HT concentrations may exceed physiologically achievable levels; difficult to distinguish HT effects from those of its metabolite	D’Angelo et al., 2005 [113]
In vitro; pure HT	25, 50, 100 μM	18 h pre-treatment; 20 min after UVB exposure	HaCaT human keratinocytes	UVB: 45 mJ/cm^2^ for 9 min; HT administered post-UVB	↓ UVB-induced genotoxicity ↓ DNA strand breaks (100 μM) ↓ ROS (~47%); ↓ 8-OHdG ↓ UVB-induced NF-κB and p53 activation	Acute UVB exposure and short-term treatment; no repeated UV exposure or long-term photoprotection; concentrations may not be achievable through diet	Guo et al., 2010 [109]
In vitro; pure HT and biocatalytically synthesized HT dimer	HT: 200–1000 μM for UVA experiments; HT dimer: 200–500 μM; broader cytotoxicity screening up to 3000 μM	24–72 h; 18 h pretreatment before UVA; 2 h post-UVA recovery for protein analyses	HaCaT human keratinocytes	UVA: 315–400 nm, 22.3 J/cm^2^	↓ ROS Protection against UVA-induced apoptosis (↑ Bcl-2, ↓ Bax/AIF) ↑ antioxidant capacity and protection from UV with HT dimer	Single immortalized keratinocyte line; relatively high concentrations; no chronic photoageing assessment	Zwane et al., 2012 [97]
In vitro; phenol-enriched purified OMWW extract containing HT and other phenolics	HT in extract: 2.52 g/L; HT glycoside: 2.01 g/L; also Verb, tyrosol derivatives, OLE/aglycone and other phenolics	24 h, 72 h, 12 days	HaCaT keratinocytes, NHEK normal human epidermal keratinocytes, A375 melanoma cells, and 3D melanoma skin model	TNF-α (10 ng/mL) co-treatment for 72 h OMWW pretreatment for 30 min followed by H_2_O_2_ (100 μM) for 45 min	↓ ROS and IL-8 Broad antimicrobial activity ↑ proliferation of normal keratinocytes ↓ melanoma nodule growth in 3D skin model	HT not evaluated as an isolated compound	Schlupp et al., 2019 [114]
In vitro; pure HT	50–500 μM; viability: 50–250 μM; mechanistic studies: 250–500 μM in A375 and 250–450 μM in HT-144	24, 48, and 72 h, depending on endpoint	A375, M74 and HT-144 human melanoma cell lines	No external stimulus	↓ melanoma-cell viability ↑ apoptosis (↑ p53, caspase-9/-3, PARP cleavage, γH2AX) ↓ Akt HT acted as a pro-oxidant (↑ ROS → oxidative DNA damage → apoptosis) ↓ colony formation	High concentrations may not be achievable through dietary intake; no normal melanocytes or skin tissue	Costantini et al., 2020 [115]
In vitro; pure HT	100 and 200 μM	48 h	Human cutaneous melanoma cell lines A375 (malignant) and MNT1 (metastatic); both BRAF V600E-mutated	No external stimulus	↓ A375 viability MNT1 cells largely resistant ↑ SNAT1 and xCT ↓ EAAT3 in A375 ↓ JNK expression and ERK phosphorylation ↓ LDHA/LDHC in MNT1 greater efficacy in glycolytic/JNK-active melanoma phenotype	Only two melanoma cell lines, both carrying BRAF V600E; concentrations may exceed dietary exposure	Brito et al., 2021 [116]
In vitro; pure OLE, HT and Verb, alone or combined	OLE: 20 μM; HT: 20 μM; Verb: 20 μM; combination: 10 μM each	24 h pretreatment before UVB, followed by additional 2–24 h treatment after UVB	Human dermal fibroblasts, HaCaT keratinocytes, and keratinocyte–fibroblast co-culture	UVB: 80 mJ/cm^2^	Combination more effective than individual compounds ↓ 8-oxo-dG, apoptosis, inflammatory cytokines, MMP-1/MMP-9, collagen degradation and MAPK/NF-κB ↓/delayed senescence ↑ Nrf2, collagen synthesis and viability	Acute UVB exposure; co-culture does not fully reproduce human skin architecture	Wang J. et al., 2025 [111]
In vitro and in vivo; pure HT	In vitro: 10, 20 and 40 μM; In vivo: 25, 50 and 100 mg/kg/day, oral gavage	In vitro: 24 h; In vivo: 8 weeks total, with UV irradiation during the final 6 weeks	In vitro: human HaCaT keratinocytes; In vivo: male BALB/c nude mice (6–7 weeks; n = 10/group)	In vitro: UVA 1.0 J/cm^2^ + UVB 20 mJ/cm^2^; In vivo: UVA 2.86 J/cm^2^ + UVB 56 mJ/cm^2^	In vitro: ↓ oxidative stress, ROS and inflammatory mediators ↓ apoptosis and MMPs ↓ collagen/elastin degradation ↑antioxidant defenses, cell migration/proliferation and skin barrier/hydration markers In vivo ↑ skin hydration, elasticity and collagen density ↓ TEWL and improved skin integrity ↓ PI3K-AKT, MAPK, JAK-STAT and NF-κB signaling	Single cell line; nude-mouse model limits direct translation to humans; relatively high experimental HT doses	Wang E. et al., 2026 [112]
In vitro; pure HT	1–100 ppm	24–72 h; 5–11 days	3D C32 melanoma spheroids and HEMa normal melanocytes	No external stimulus	↓ melanoma spheroid growth and viability (IC_50_ 51.1 ppm), with lower toxicity toward melanocytes (IC_50_ 105.7 ppm) ↓ migration, invasion and EMT ↑ cell-cycle arrest/apoptosis ↓ PI3K-Akt, MAPK/ERK, VEGFR-2 and ErbB2/3/4 signalling	One melanoma and one normal melanocyte model; concentrations may not be achievable through dietary intake; no long-term efficacy/safety in vivo	Tovar-Parra & Mangion, 2025 [117]
Review of preclinical in vitro 2D melanoma studies of pure HT and OLE	50–1000 µM	Primarily 24, 48 and 72 h	Human melanoma cell lines: C32 (amelanotic), A375, MNT1, HT-144 and M74	No additional external stimulus	Broad anticancer activity ↓ proliferation, oxidative stress, Akt, NF-κB, MAPK, HIF-1α, angiogenesis, invasion and metastasis ↑ cell-cycle arrest and apoptosis	Limited clinical evidence; concentrations may not be achievable through dietary consumption; heterogeneous protocols, doses, formulations and cancer models	Gervasi et al., 2024 [118]

Abbreviations: 3D, three-dimensional; 8-OHdG, 8-hydroxy-2′-deoxyguanosine; AIF, apoptosis-inducing factor; Akt, protein kinase B; Bax, Bcl-2-associated X protein; Bcl-2, B-cell lymphoma 2; EAAT3, excitatory amino acid transporter 3; ERK, extracellular signal-regulated kinase; HaCaT, human adult low-calcium high-temperature keratinocytes; γH2AX, phosphorylated histone H2AX; LDHA, lactate dehydrogenase A; LDHC, lactate dehydrogenase C; HEMa, human epidermal melanocytes; HT, hydroxytyrosol; JNK, c-Jun N-terminal kinase; MAPK, mitogen-activated protein kinase; MMP-1, matrix metalloproteinase-1; NF-κB, nuclear factor kappa B; NHEK, normal human epidermal keratinocytes; Nrf2, nuclear factor erythroid 2-related factor 2; OMWW, olive mill wastewater; PARP, poly(ADP-ribose) polymerase; PI3K, phosphoinositide 3-kinase; ROS, reactive oxygen species; SNAT1, sodium-coupled neutral amino acid transporter 1; TBARS, thiobarbituric acid reactive substances; UVA, ultraviolet A; UVB, ultraviolet B; VEGFR-2, vascular endothelial growth factor receptor 2; xCT, cystine/glutamate antiporter.

### 6.2. Skin Cancer Prevention

Evidence supporting a potential role of HT in skin cancer prevention is predominantly derived from preclinical melanoma models (Table 2). Collectively, these studies suggest that HT may exert both protective effects against UV-induced molecular damage and direct antitumor activity, although its biological effects appear strongly dependent on concentration and cellular context.

Early evidence showed that HT could protect melanoma cells against UVA-induced oxidative damage while, at higher concentrations, simultaneously exerting antiproliferative and pro-apoptotic effects [113]. This concentration-dependent behaviour is particularly relevant because it illustrates the dual biological activity of HT: at lower or moderate exposures it may primarily act as an antioxidant, whereas at higher concentrations it may promote oxidative stress and tumour-cell death.

Subsequent studies strengthened the evidence for direct antitumor activity. An HT-containing olive mill wastewater extract reduced melanoma growth in a three-dimensional skin model while maintaining favourable effects in normal keratinocytes, although the contribution of HT itself cannot be distinguished from that of the other phenolic constituents [114]. Studies using purified HT further demonstrated inhibition of melanoma-cell survival through pro-apoptotic and pro-oxidant mechanisms involving modulation of p53, Akt, DNA-damage responses, and other survival pathways [115].

Importantly, melanoma susceptibility to HT does not appear to be uniform. Comparative experiments showed substantially different responses between melanoma cell lines, suggesting that metabolic phenotype may influence sensitivity to HT. In particular, alterations in amino-acid transport, glycolytic metabolism, and JNK/ERK signalling indicate that metabolic reprogramming may contribute to its antitumor activity [116]. More recent evidence from three-dimensional melanoma spheroids further supports this concept, showing preferential activity against melanoma cells relative to non-tumorigenic melanocytes and identifying multiple oncogenic signalling networks as potential targets [117]. These findings suggest that HT may act through a multitarget mechanism rather than through a single anticancer pathway.

Despite these promising findings, the translational relevance of the available evidence remains uncertain. As emphasized by Gervasi et al., the concentrations producing anticancer effects in experimental systems frequently exceed those achievable through dietary intake, and the biological activity of HT may shift between antioxidant and pro-oxidant effects depending on dose and cellular context [118]. Bioavailability and metabolism therefore represent major considerations when extrapolating in vitro findings to humans.

Overall, current evidence supports the biological plausibility of HT as a photoprotective and potential chemopreventive agent but remains predominantly preclinical. Future studies should determine whether effective concentrations can be achieved safely in skin tissue, clarify the influence of melanoma phenotype on treatment response, and establish whether optimised topical or transdermal delivery systems can overcome current pharmacokinetic limitations.

The evidence for HT photoprotective and anticancer effects is summarized in Table 2.

## 7. Translational Challenges and Study Limitations

Despite the promising mechanistic and preclinical evidence, several challenges currently limit the translation of HT into evidence-based dermatological applications. First, the available evidence remains relatively limited and is predominantly derived from in vitro studies and animal models. Although these investigations provide valuable mechanistic insights into the antioxidant, anti-inflammatory, and regenerative properties of HT, their findings cannot be directly extrapolated to humans because of differences in skin physiology, metabolism, dosing regimens, and experimental models.

Second, the current clinical evidence is scarce and highly heterogeneous. Published studies differ considerably in participant characteristics, HT source (synthetic HT versus olive-purified HT versus HT-rich olive-derived formulations), formulation, dosage, treatment duration, comparator groups, and outcome measures. In addition, many commercially available or experimental formulations contain other bioactive olive phenols, including tyrosol, Verb, and secoiridoids, making it difficult to distinguish the specific contribution of HT from the potential synergistic effects of the phytocomplex. These methodological differences limit direct comparisons among studies and currently preclude robust quantitative synthesis.

Another important challenge concerns the selection of clinically relevant endpoints. Most human studies have primarily investigated systemic antioxidant, anti-inflammatory, or cardiometabolic outcomes rather than validated dermatological measures. Consequently, relatively few clinical trials have objectively assessed skin-specific parameters such as hydration, elasticity, transepidermal water loss, wrinkle morphology, dermal thickness, collagen integrity, pigmentation, or wound-healing outcomes, making it difficult to establish clear relationships between the molecular mechanisms described in preclinical models and clinically meaningful improvements in skin health.

Finally, although this review discusses the recovery of HT from OMWW within the framework of sustainability and the circular economy, it does not provide a comparative evaluation of extraction technologies, manufacturing scalability, regulatory aspects, economic feasibility, or life-cycle environmental impacts. These topics deserve dedicated investigation as HT-rich formulations continue to be developed for nutraceutical and dermatological applications.

Future research should therefore prioritise adequately powered randomised controlled trials employing standardised HT formulations, comprehensive pharmacokinetic and dose–response assessments, validated skin biomarkers, microbiome-related parameters, and clinically relevant dermatological endpoints. The use of advanced experimental models, such as three-dimensional reconstructed human skin and skin organoids, together with long-term clinical studies, will be essential to clarify the relationship between the molecular effects of HT and clinically meaningful improvements in cutaneous homeostasis, healthy ageing, and skin disease prevention.

## 8. Future Directions: Emerging Mechanisms and Research Priorities

Beyond its established antioxidant, anti-inflammatory, and regenerative properties, HT may exert additional biological activities relevant to skin health, although the available evidence remains largely exploratory.

One area of growing interest concerns the potential interaction between HT and oestrogen-related pathways. Due to structural similarities with estradiol, HT has been proposed to exhibit phytoestrogen-like activity, potentially influencing oestrogen receptor signalling [119,120]. This hypothesis is particularly intriguing in the context of menopause, where oestrogen decline contributes to reduced collagen synthesis, impaired skin barrier function, loss of elasticity, and accelerated skin ageing. In a randomised, double-blind, placebo-controlled clinical trial involving 60 postmenopausal women, supplementation with olive leaf extract significantly improved menopause-related quality of life, increased bone mineral density, and improved selected cardiometabolic parameters [120]. However, because the intervention consisted of a complex olive-derived extract rather than purified HT and skin-related outcomes were not specifically evaluated, further studies are required to address the potential role of HT in menopause-associated skin ageing.

Another emerging field concerns the possible contribution of HT to skin and gut microbiome homeostasis. A growing body of preclinical evidence suggests that HT and HT-derived compounds possess antimicrobial and anti-biofilm properties against a variety of bacterial species. In vitro and in silico studies have shown that HT extracted from olive leaves exhibits antibacterial activity against both standard and extended-spectrum β-lactamase (ESBL)-producing bacteria through interactions with essential bacterial proteins involved in DNA replication and cell wall synthesis [121]. Similarly, HT-based polymers have demonstrated antioxidant, antibacterial, and anti-adhesive activity against Staphylococcus epidermidis, reducing bacterial adhesion and biofilm formation in vitro [122]. HT acetate has also shown antibacterial activity against Vibrio species through mechanisms involving increased membrane permeability and disruption of bacterial DNA function [123].

Although these findings suggest additional biological activities of HT, evidence is currently limited to experimental models. Their relevance to skin physiology, the gut–skin axis, and healthy ageing therefore requires confirmation in well-designed mechanistic and clinical studies.

## 9. Discussion

This review summarizes the current evidence regarding the potential role of HT and HT-rich olive-derived formulations in skin health and healthy ageing. The main contribution of this review is the integration of dispersed evidence from redox biology, skin ageing, nutraceutical research, formulation science, and dermatological studies into a skin-focused framework for oral and topical HT and HT-rich olive-derived products. Overall, the available literature suggests that HT exerts multiple biological activities that are highly relevant to skin homeostasis, including antioxidant, anti-inflammatory, photoprotective, and regenerative effects. These mechanisms target several key drivers of skin ageing, such as oxidative stress, chronic low-grade inflammation, ECM degradation, cellular senescence, and impaired tissue repair [40,52]. This topic is timely given the growing interest in redox-active nutraceuticals, dietary strategies for skin ageing, and the sustainable valorization of olive-derived by-products.

However, one of the most intriguing aspects emerging from the current evidence is the apparent discrepancy between the pharmacokinetic characteristics of HT and its broad spectrum of biological activities. Despite being rapidly absorbed, extensively metabolized, and exhibiting relatively low circulating concentrations of free HT, consistent biological effects have been demonstrated across mechanistic, preclinical, and, to a lesser extent, clinical studies. This suggests that the biological efficacy of orally administered HT cannot be explained solely by the parent compound but rather by the combined contribution of its metabolites, endogenous antioxidant responses, and formulation-dependent pharmacokinetics.

Overall, the available mechanistic and preclinical evidence provides a coherent biological rationale for the use of HT in skin health. Across different experimental models, HT consistently modulates key pathways involved in skin ageing, including oxidative stress, inflammatory signalling, ECM remodelling, cellular senescence, and tissue repair [94,95,96,99], while animal studies further support its ability to preserve skin structure and function [52,104]. The strength of evidence differs across outcomes, with the most consistent support coming from cellular, reconstructed skin, ex vivo, and animal studies, whereas direct human dermatological evidence remains preliminary.

Importantly, the biological activity of HT extends beyond direct free-radical scavenging. Although its catechol structure confers potent antioxidant properties, increasing evidence indicates that HT primarily acts by modulating endogenous cytoprotective pathways, including Nrf2-mediated antioxidant defences, NF-κB inhibition, PI3K/Akt and MAPK signalling, autophagy, and mitochondrial function, thereby regulating cellular redox homeostasis. This activity is likely supported by HT metabolites, including glucuronide and sulphate conjugates, which retain biological activity or may serve as circulating reservoirs capable of releasing active HT within tissues [68].

Recent experimental studies suggest that HT protects against UV-induced skin damage and may contribute to skin cancer prevention by reducing oxidative DNA damage, inflammation, cellular senescence, and apoptosis, while inhibiting melanoma cell proliferation and angiogenic signalling through modulation of PI3K/Akt, MAPK/ERK, and VEGF pathways [109,114,115,116,117]. Although currently limited to experimental models, these findings broaden the potential dermatological applications of HT and warrant further translational investigations.

An important determinant of HT efficacy is its formulation. Owing to its hydrophilic nature and rapid metabolism, HT bioavailability and tissue exposure may be substantially influenced by delivery systems, including HT-rich phytocomplexes, lipid-based carriers, and encapsulation technologies. These formulations may improve chemical stability, absorption, and systemic persistence, thereby enhancing biological efficacy. This consideration is particularly relevant when interpreting studies employing HT-rich olive-derived formulations. While clinical studies consistently report improvements in oxidative stress, inflammation, and selected cardiometabolic parameters [31,85,88,89,91,92], these products frequently contain additional bioactive phenolics, including tyrosol, Verb, and secoiridoids. Therefore, their biological effects likely result from both the activity of HT and synergistic or pharmacokinetic interactions among multiple compounds. Distinguishing true molecular synergy from formulation-dependent improvements in bioavailability remains an important challenge for future research.

Beyond its direct effects on the skin, HT may indirectly promote skin health through improvements in vascular function, metabolic homeostasis, oxidative balance, and chronic low-grade inflammation [34,40]. Emerging evidence also supports a role for HT in modulating gut microbiota composition and intestinal barrier integrity, reinforcing the concept of the gut–skin axis [61,62].

Despite this strong biological rationale, only a few clinical studies have evaluated dermatological outcomes, mainly in psoriasis and melasma [106,107]. Moreover, the available trials are characterized by small sample sizes, heterogeneous formulations, variable dosages, short treatment durations, and limited use of validated dermatological endpoints, restricting the strength and generalisability of current conclusions. The evidence base also spans different experimental systems, including cell-based models, reconstructed skin models, animal models, and human studies, which limits direct comparability across studies. Additional areas deserving further investigation include wound healing, microbiome modulation, inflammatory skin diseases, UV-induced photoageing, melanoma prevention [99,105,121], and the potential phytoestrogen-like activity of HT in postmenopausal skin ageing [119,120]. This review also has inherent limitations. As a narrative review, it integrates mechanistic, preclinical, and clinical evidence characterized by substantial heterogeneity in study design, formulations, and outcome measures. Consequently, the overall strength of evidence varies across research levels and should be interpreted accordingly.

Future studies should focus on elucidating the relationships among HT formulation, metabolism, tissue exposure, and clinical response. Comparative investigations of purified HT, its metabolites, HT-rich phytocomplexes, and advanced delivery systems, combined with pharmacokinetic analyses and validated dermatological endpoints, will be essential to improve the translational relevance of this field. Future dermatological trials should include standardised HT dose reporting, detailed phenolic characterization of formulations, adequate treatment duration, and validated outcomes such as skin hydration, barrier function, clinical severity scores, pigmentation or erythema indices, dermal imaging, and biomarkers of oxidative stress and inflammation.

Overall, current evidence supports HT as a promising nutraceutical capable of modulating multiple pathways involved in skin homeostasis and healthy ageing. Rather than acting solely as a direct antioxidant, HT should be regarded as a pleiotropic redox-modulating molecule whose efficacy depends on the interplay among its chemical properties, metabolism, formulation, and endogenous cellular defence mechanisms. Although additional well-designed clinical trials are needed, the available evidence provides a solid scientific rationale for the continued development of HT-based nutraceuticals and sustainable olive-derived formulations for skin health and healthy ageing.

## 10. Conclusions

HT is a promising olive-derived polyphenol with antioxidant, anti-inflammatory, and photoprotective properties that may contribute to the maintenance of cutaneous homeostasis and healthy ageing. Current mechanistic and preclinical evidence consistently supports its ability to modulate oxidative stress, inflammation, ECM remodelling, cellular senescence, and tissue repair, while preliminary clinical studies suggest potential benefits in selected dermatological conditions.

The current evidence is strongest for biological plausibility and preclinical activity, whereas clinically meaningful dermatological benefits require further confirmation. Future well-designed randomised controlled trials using standardised formulations and validated dermatological endpoints are needed to clarify the relationship between the biological effects of HT and clinically meaningful improvements in skin health. Overall, the current evidence supports HT as a promising nutraceutical for dermatological applications, while further clinical validation is required before its routine use can be recommended.

## Figures and Tables

**Table 1:** Preclinical evidence supporting the protective effects of hydroxytyrosol on skin homeostasis and ageing-related pathways.

Type of Study and Intervention	Concentration/Dose	Experimental Period	Cell/Model Type	Stimulus/Experimental Condition	Main Findings	Limitations	Reference
In vitro; pure HT (>90%)	5 and 10 μM	24 h	Human dermal fibroblasts	Cells pre-treated for 6 h before UVA irradiation (8 J/cm^2^)	↓ cellular senescence (↓ SA-β-gal) ↓ MMP-1, MMP-3, IL-1β, IL-6, and IL-8	Short-term exposure; single cell line; no protein or functional analyses	Jeon & Choi, 2018 [95]
In vitro; pure HT, alone or combined with OLE (1:1 ratio)	HT: 12.5–1000 μM	24 h	Human dermal fibroblasts	H_2_O_2_-induced oxidative stress cytotoxicity assay: 300 μM for 24 h ROS level: HT treatment 24 h, followed by 100 μM H_2_O_2_ for 1 h	Concentration-dependent inhibition of collagenase (5.8–12.2%) and elastase (11.6–31.9%); uncompetitive elastase inhibition ↓ H_2_O_2_-induced cytotoxicity (up to 32.0%) ↓ ROS (up to 15.2%). HT/OLE: ↓ elastase activity ↓ ROS (7.6–37.3%)	Short-term exposure; moderate enzyme inhibition mainly at high concentrations; low skin permeability predicted	Li et al., 2022 [102]
In vitro; pure HT	1 μM	Chronic treatment, 4–6 weeks	Pre-senescent MRC5 human lung fibroblasts and neonatal human dermal fibroblasts	Pre-senescent conditions; TNF-α 10 ng/mL for 3 h used for inflammatory activation	Delayed cellular senescence ↑ cell number ↓ SA-β-gal-positive cells ↓ p16, SASP, IL-6 ↓ MMP-2/MMP-9 activity ↓ NF-κB activation ↓ COX-2, α-SMA, and TNF-α-induced inflammatory activation	Mixed cell types, including non-cutaneous fibroblasts	Menicacci et al., 2017 [96]
In vitro; pure HT and HT acetate (HT-Ac)	12.5–100 μM	30-min pretreatment; stimulation for 15 min–4 h	Primary human keratinocytes	IL-1β or TLR3 ligand stimulation Inflammatory stimuli: -IL-1β, 10 ng/mL -TLR3 ligand (Poly I:C), 10 μg/mL	↓ NF-κB-dependent transcription ↓ TSLP, IL-8, IL-6, TNF-α, and COX-2	Possible donor variability; acute inflammatory model	Aparicio-Soto et al., 2019 [94]
In vitro; pure HT	25–200 μM; 50 μM for mechanistic experiments	2-h pretreatment; M5 exposure for 48 h	HaCaT keratinocytes	M5 cytokine cocktail: TNF-α, IL-17A, IL-22, IL-1α, and oncostatin M	↓ IL-6, IL-8, TNF-α, and psoriasis-associated antimicrobial proteins (hBD-2, S100A7, S100A8, S100A9) ↓ keratinocyte proliferation ↑ G1/S cell-cycle arrest	Immortalized HaCaT cells; molecular mechanisms not investigated; no protein-level validation	Chen et al., 2023 [103]
In vitro; pure HT and biocatalytically synthesized HT dimer	HT: 200–1000 μM; HT dimer: 200–500 μM	18-h pretreatment before UVA exposure; 2-h post-UVA recovery	HaCaT keratinocytes	UVA exposure: -wavelength 315–400 nm -dose 22.3 J/cm^2^	↑ antioxidant activity ↑ resistance to UV-induced apoptosis (↑ Bcl-2 and ↓ Bax) Greater effect with HT dimer	No primary skin cells; relatively high HT concentrations	Zwane et al., 2012 [97]
In vitro; pure HT	10–100 μM; 50 μM for mechanistic experiments	0–24 h time course; 10–100 μM HT: 3 h for HO-1 mRNA and 24 h for HO-1 protein	Porcine vascular endothelial cells	Wound-healing/endothelial repair model	↑ Nrf2 → ↑ HO-1 through PI3K/Akt and ERK1/2 ↑ wound closure through HO-1-derived carbon monoxide	Non-human and non-skin cells; wound healing assessed in endothelial monolayers; no collagen synthesis evaluation	Zrelli et al., 2015 [100]
In vitro; pure HT	0.025–2% for cytotoxicity screening; 0.2% and 0.4% for functional studies	24–48 h for migration; 5 days for EMT markers	Human dermal fibroblasts	EMT induced with TGF-β 5 ng/mL for antifibrotic assessment	↑ fibroblast proliferation, migration, and wound closure, with maximal effects at 0.2% and 0.4% ↑ antifibrotic effect through inhibition of TGF-β-induced EMT	Small number of donor samples (n=5); short-term exposure	Batarfi et al., 2023 [98]
In vitro; pure HT	10^−5^–10^−9^ M; functional assays mainly at 10^−5^ and 10^−6^ M	24 h	CCD-1064Sk human skin fibroblasts	Wound-closure/migration model	↑ fibroblast proliferation and migration; earliest and greatest wound closure among tested polyphenols, with complete closure after 24 h ↑ fibronectin and α-SMA	No primary cells; short-term exposure	González-Acedo et al., 2023 [99]
In vivo; pure oral HT	25 or 50 mg/kg/day	16 weeks	Male C57BL/6J mice	Dietary AGE-induced skin ageing: AIN-93G-based feed, heat-treated at 128 °C for 3 h	↑ skin hydration, epidermal and dermal thickness, and hydroxyproline ↓ oxidative stress (↑ SOD, ↑ GSH-Px, ↓ MDA) ↓ systemic IL-1β, IL-6, and TNF-α ↑ intestinal barrier integrity (↑ ZO-1 and occludin)	Animal study; high HT dose	Fan et al., 2025 [52]
In vitro and in vivo; pure HT	In vivo: 10–50 mg/kg/day In vitro: 50 and 100 μM	In vivo: HT 3 days → HT + IMQ 6 days → sacrifice day 10 In vitro: HT 2 h → M5 cytokine stimulation	Female BALB/c mice HaCaT keratinocytes	In vivo: psoriasis-like dermatitis induced with 62.5 mg/day IMQ In vitro: M5 cytokine stimulation	↓ PASI scores, epidermal hyperplasia, acanthosis, inflammatory infiltration, PCNA, and cutaneous/systemic cytokines ↓ splenomegaly and immune-cell infiltration ↓ ERK and NF-κB activation ↓ keratinocyte hyperproliferation and inflammatory responses	No investigation of oxidative stress/antioxidant mechanisms; acute IMQ model; immortalized keratinocytes; limited effects on differentiation	Liu et al., 2025 [104]
In vitro and in vivo; pure HT; separate EVOO-enriched diet comparison	100 μM HT	Applied to wounds for 3 consecutive days In vitro: 8–96 h depending on the cellular endpoint, and up to 11 days for collagen-gel contraction	Streptozotocin-induced diabetic mice; 3T3 mouse fibroblasts and RAW264.7 macrophages	Cutaneous wounds under diabetic conditions In vitro high-glucose conditions (48 mM)	↑ wound healing, re-epithelialization, collagen deposition, fibroblast migration and contraction, M2 macrophage polarization and IL-10 ↓ oxidative stress and inflammation ↑ HO-1/Nrf2 signalling	Diabetic murine model and immortalized cells; limited direct extrapolation to human skin ageing	Duarte et al., 2024 [105]

Abbreviations: Akt, protein kinase B; Bax, Bcl-2-associated X protein; Bcl-2, B-cell lymphoma 2; CCD-1064Sk, human skin fibroblast cell line; COX-2, cyclooxygenase-2; EMT, epithelial-to-mesenchymal transition; ERK1/2, extracellular signal-regulated kinases 1 and 2; EVOO, extra virgin olive oil; GSH-Px, glutathione peroxidase; HaCaT, human adult low-calcium high-temperature keratinocyte cell line; HO-1, heme oxygenase-1; HT, hydroxytyrosol; H_2_O_2_, hydrogen peroxide; IL, interleukin; IMQ, imiquimod; M5, cytokine cocktail composed of TNF-α, IL-17A, IL-22, IL-1α, and oncostatin M; MDA, malondialdehyde; MMP, matrix metalloproteinase; NF-κB, nuclear factor kappa B; Nrf2, nuclear factor erythroid 2-related factor 2; OLE, oleuropein; PASI, Psoriasis Area and Severity Index; PI3K, phosphoinositide 3-kinase; ROS, reactive oxygen species; SA-β-gal, senescence-associated β-galactosidase; SASP, senescence-associated secretory phenotype; SOD, superoxide dismutase; α-SMA, α-smooth muscle actin; TLR3, Toll-like receptor 3; TNF-α, tumor necrosis factor alpha; TSLP, thymic stromal lymphopoietin.

## Data Availability

No new data were created or analysed in this study. Data sharing is not applicable to this article.
